# High Frequency Ultrasound of Basal Cell Carcinomas: Ultrasonographic Features and Histological Subtypes, a Retrospective Study of 100 Tumors

**DOI:** 10.3390/jcm12123893

**Published:** 2023-06-07

**Authors:** Styliani Siskou, Paola Pasquali, Myrto Trakatelli

**Affiliations:** 1 Second Dermatology Department, School of Medicine, Faculty of Health Sciences, Aristotle University of Thessaloniki, 54124 Thessaloniki, Greece; mtrakatelli@hotmail.com; 2 Department of Epidemiology and Population Health, London School of Hygiene and Tropical Medicine, London WC1H 9SH, UK; 3 Dermatology Department, Pius Hospital de Valls, Universidad de Alacalá, 43800 Valls, Spain; ppasquali@piushospital.cat

**Keywords:** high frequency ultrasound, basal cell carcinoma, histology subtype, non-invasive imaging techniques, ultrasound

## Abstract

(1) Background: 22 MHz high frequency ultrasound (HFUS) is a non-invasive imaging technique that gives information on depth, length, volume and shape of skin tumors. (2) Methods: We reviewed the clinical, ultrasound, and histological records of 54 patients with 100 histologically confirmed basal cell carcinoma (BCC) tumors with the use of HFUS. (3) Results: Most infiltrative tumors (*n* = 16/21, 76.2%) were irregular shaped, followed by five (23.8%) being round shaped; most superficial tumors (*n* = 25/29, 86.2%) were ribbon shaped, followed by four (13.8%) being round shaped; most nodular tumors (*n* = 26/33, 78.8%) were round shaped, followed by seven (21.2%) that were irregular shaped; and, lastly, all microdular tumors (*n* = 2/2, 100%) were round shaped. Strong evidence of association (*p* = 0.000) was observed between the histological subtype and tumor shape as seen using the HFUS. No evidence of association was found between the histological subtype and tumor margin (*p* > 0.005). Cohen’s Kappa statistic to assess the agreement between BCC subtypes evaluated by histological examination and U/S appearance was calculated equal to 0.8251 (almost perfect agreement). (4) Conclusions: HFUS appears to be a reliable technique for the pre-operative evaluation of BCCs, assisting physicians to decide on the optimal therapeutic approach.

## 1. Introduction

Basal cell carcinoma (BCC) is the most common type of skin cancer, comprising 75% to 80% of all types, and it is the most common malignant tumor in white populations [1,2]. Due to changes in sun exposure habits, as well as due to an increase in the life span of people in western societies, the incidence of these tumors is continuously on the rise. Furthermore, BCCs in general have low mortality, but as they mainly occur on the head and neck area, their morbidity is significant and can create severe impairments, especially when they are not treated in timely fashion and have acquired a significant size [3]. Diagnosis is commonly achieved by a combination of clinical and dermatoscopic findings, which tend to be useful in the preoperative prediction of the BCC subtype. Determining the non-invasive potential of a tumor helps us to predict the response to topical or minimally invasive treatments. The sensitivity and specificity of dermoscopy is higher for pigmented than non-pigmented BCCs and when performed by experts [2]. Furthermore, tumors located in difficult-to-treat locations (eyes, nose, lips, ears) and those with poorly defined non-pigmented margins which are often associated with the morphoeic subtype or recurrent tumors pose a diagnostic challenge because an accurate appreciation of the margins is often impossible. We need other non-invasive imaging options that will allow us to predict the exact nature of the tumor.

Early, accurate detection of skin cancer is essential to guide appropriate management and to improve morbidity and quality of life [4]. Additional tools should be used to enhance diagnostic accuracy. Diagnostic techniques, such as optical coherence tomography (OCT), reflectance confocal microscopy (RCM), and ultrasound, have been proposed and studied for basal cell carcinoma management [2]. Cutaneous high-frequency ultrasound examinations can accurately and rapidly differentiate between epidermal, subdermal, and subcutaneous tissues in real time. This procedure may help to identify lesions invisible to the spatially-restricted human eye [5].

High Frequency Ultrasound (HFUS) is a non-invasive, office-based technique that can be used to detect the optimal biopsy site and the depth, the length, and morphology (volume/shape) of the tumor, and also to differentiate BCCs from other skin tumors and, possibly, differentiate different BCC histological subtypes [6,7,8]. Different tumor subtypes behave in a different manner, so the need to recognize these subtypes becomes all the more relevant. In addition, not all tumors need to be treated with surgery: there are many minimally and non-invasive techniques that can be used on low-risk tumors, and the therapeutic choice also depends on the tumor’s characteristics (tumor size, subtype, etc.).

In dermatology, high resolution devices with high frequency transducers are used [9,10]. Devices of 20 to 25 MHz are most frequently used, and have the best resolution for the observation of surface structures. Frequencies between 50 and 100 MHz present little penetration, limited to the epidermis [7,10]. Apart from HFUS application to skin tumors, HFUS can also be used to examine other skin conditions, such as inflammatory and infectious cutaneous diseases, skin aging, and cosmiatry [10].

In our study, we aim to explore the value of HFUS in identifying high-risk BCC tumors and in differentiating the latter from low-risk ones that require treatment with minimally invasive techniques, and we also aim to correlate tumor characteristics as they appear in ultrasound (tumor depth, shape, morphology of margins) with the various histological subtypes. HFUS can prove to be an addition to dermoscopy as a non-invasive diagnostic tool that will enhance our diagnostic accuracy in skin oncology, and that will also guide us through therapeutic decision making.

## 2. Materials and Methods

For the aims of this study, we retrospectively reviewed clinical, ultrasound, and histological records of patients with 100 basal cell carcinomas, diagnosed from 26 May 2016 to 11 November 2016 at the Dermatology Department of the Pius Hospital of Valls in Spain. A total of 54 patients (35 men, 19 women) with 100 basal cell carcinomas diagnosed clinically and dermoscopically [11] were included in the study. Every tumor was initially recorded using a dermoscope Fotofinder leviacam^®^ (FotoFinder systems GmbH, Bad Birnbach, Germany) and ultrasound imaging was afterwards performed with Dub^®^Skin Scanner (Taberna ProMedicum GmbH, Lueneburg, Germany) using a 22 MHz transducer. All tumors were then removed surgically, immediately fixed with 10% formaldehyde solution, and sent for histologic evaluation. Hematoxylin and eosin dyes were used for specimen staining. The samples were evaluated by the Pathology Department at the Pius Hospital of Valls, Spain. The same dermatologist performed the clinical, dermoscopic, and ultrasonographic examination, as well as the surgical excision of the tumor (PP). HFUS tumor measurements were taken during the evaluation.

### 2.1. Evaluation of Ultrasound Images

In HFUS images, the epidermis and dermis appear as a hyperechoic layer (bright) with the dermis showing up less bright than the epidermis. The subcutaneous tissue appears hypoechoic with hyperechoic fibrous septa in between. Basal cell carcinomas appear hypoechoic in contrast to the adjacent healthy tissue, while the margins can be delimited based on the difference in refraction between the area and the hyperechoic perilesional region [12]. In addition, with ultrasound imaging, it is possible to assess the tumor volume, length, and width, as well as the layers it infiltrates.

**Figure 1:**
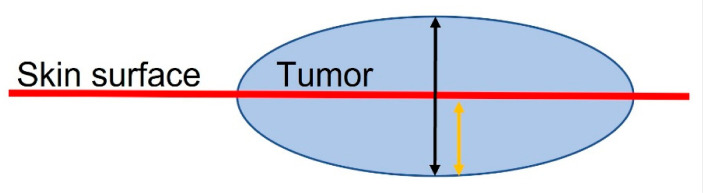
The red line represents the skin surface, and the blue sphere represents a BCC. The black arrow corresponds to tumor depth as obtained by HFUS before tumor debulking (‘HFUS depth’ variable), whereas the yellow arrow corresponds to tumor depth as obtained by HFUS after debulking (‘HFUS skin’ variable).

**Figure 2:**
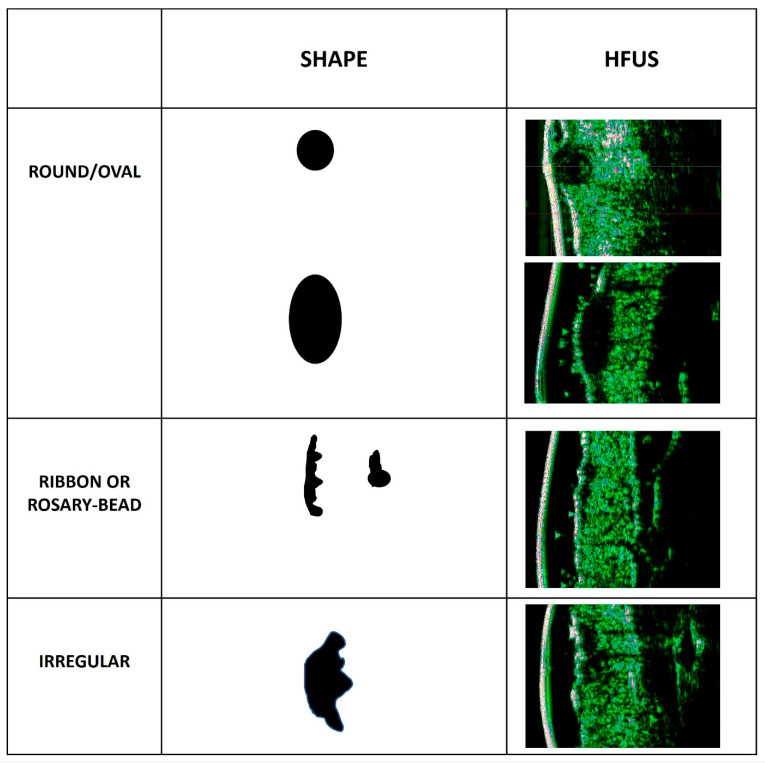
BCC shapes as observed with 22 MHz High Frequency Ultrasound. The two columns on the left describe and illustrate the tumor shape, and the right column provides HFUS images in correlation to the shapes mentioned.

In our study, one dermatologist (PP) trained in skin imaging and blinded to the patients’ histopathological results evaluated the ultrasonographic features of the lesions, including the tumour shape, margin, hyperechoic spots, width, and depth (measured in mm). Tumor depth measurements were obtained from the epidermal level and also from the surface level after subtracting the exophytic part of the tumour. Figure 1 illustrates how the measurements were taken:

The tumour ultrasound characteristics were described as in Wang SQ et al. [13]. The selected tumour shape categories included were round/oval, ribbon-like (rosary-beads like), and irregular shapes. Tumour margins were described as either well or ill defined. Figure 2 shows some examples of the most common types of BCC shapes found in the HFUS images. Ribbon/Rosary-bead tumors can be seen as an elongated thin hypoechoic strip or sometimes as a tiny round tumor with a barely visible elongation. Irregular tumors can have very diverse shapes.

### 2.2. Statistics

The statistical package STATA version 14.2 (StataCorp, College Station, Texas, USA) was used for data analysis. The analysis comprised a preliminary descriptive analysis assessing the main characteristics of our sample population. Subsequently, we stratified our dataset according to BCC subtype, and HFUS measurements were calculated for each category. Absolute and relative frequencies of tumor shape and margins were obtained for the various subtypes. Cross-tabulations and hypothesis testing using the *p*-value approach was used to examine possible associations. One-way analysis of variance (ANOVA) was used to determine whether there were any statistically significant differences between the means of tumor depth for the various subtypes. Cohen’s Kappa coefficient (κ) was used to measure inter-rater reliability.

## 3. Results

### 3.1. Dataset Characteristics

From 100 tumors that were included in the study, the majority referred to male patients (*n* = 66, 66.0%) and 34 to female patients (34.0%). The mean age of the study population was 73.1 years. Most BCCs (*n* = 33, 33.0%) included in the dataset were nodular, followed by superficial (*n* = 29, 29.0%), infiltrative (*n* = 21, 21.0%), and micronodular *n* = 2 (2.0%). A total of 14 tumors (14.0%) had no indication of the histologic subtype in the histogical report and 1 was reported as superficial plus infiltrative. The majority (*n* = 66, 66.0%) of the tumors were located on the head and neck area (Table 1).

### 3.2. HFUS Tumor Features (Shape and Margins) and Correlation with BCC Histological Subtype

When assessing the tumor shape, the majority *n* = 46 (46.0%) were round shaped, followed by 28 tumors (28.0%) that were irregular shaped and 26 (26.0%) that were ribbon shaped. The vast majority (*n* = 74, 74.0%) were well-defined tumors, whereas 26 (26.0%) were ill defined. (Table 1) There was found to be some evidence of association between the tumor shape and the tumor margin (*p* = 0.013), with ribbon- and round-shaped tumors most commonly having well-defined margins. In detail, 80% of ribbon-shaped and 81% of round-shaped tumors had well-defined margins. Irregular-shaped tumors were found to have either ill- (*n* = 12, 52.2%) or well-defined (*n* = 11, 47.8%) margins. After excluding the tumors that had no indication of histology reported as well as the one that was evaluated as superficial plus the infiltrative tumor type, we stratified our dataset by histological subtype and found that most infiltrative tumors (*n* = 16/21, 76.2%) were irregular shaped, followed by 5 (23.8%) being round shaped; that most superficial tumors (*n* = 25/29, 86.2%) were ribbon shaped, followed by 4 (13.8%) being round shaped; that most nodular tumors (*n* = 26/33, 78.8%) were round shaped, followed by 7 (21.2%) that were irregular shaped; and, lastly, that all microdular tumors (*n* = 2/2, 100.0%) were round shaped (Table 2A). Strong evidence of association (*p* = 0.000) was observed between histological subtype and tumor shape, as seen with the HFUS. (Table 2A) No evidence of association was found between histological subtype and tumor margin (*p* > 0.005) (Table 2B).

As far as the positive predictive value of HFUS is concerned: (1) 76.2% of infiltrative BCCs were irregular shaped and 69.6% of all irregular-shaped tumors were found to be infiltrative BCCs, so in our dataset, when the tumor is irregular shaped, there is a 69.6% possibility of being an infiltrative BCC. (2) A total of 86.2% of superficial BCCs were ribbon shaped, and 100.0% of all ribbon-shaped tumors were found to be superficial BCCs, so in our dataset, ribbon-shaped tumors have a 100.0% probability of being superficial BCCs. (3) A total of 78.8% of nodular BCCs were round shaped and 70.3% of round-shaped BCCs were found to be nodular. Round-shaped tumors have a 70.3% probability of being nodular BCCs in our study.

### 3.3. HFUS Measurements (Tumor Depth and Length)

In regards to HFUS measurements, the mean HFUS depth was calculated as 1702 (SD = 992.6), whereas the mean depth from the surface of the skin after tumor debulk was 1180.8 (SD = 572.3). After stratifying the dataset according to BCC subtype, we calculated the tumor depth before and after tangential excision. The subject defined as superficial plus infiltrative tumor type and the ones without a histology report were all excluded from the analysis.

#### 3.3.1. Before BCC Tangential Excision, by BCC Histological Subtype

When the whole volume of the tumor was measured, the following mean depth values were obtained: micronodular tumors measured 2656.5 (SD = 613.1), nodular 2413.4 (SD = 798.2), infiltrative 1692 (SD = 791.2), and superficial 620.6 (SD = 255.5) (Table 3). A one-way analysis of variance showed that the effect of BCC subtype on tumor depth was significant, F (4,93) = 32.05, *p* = 0.000 (Table 4A).

#### 3.3.2. After BCC Tangential Excision, by BCC Histological Subtype

We remeasured the ultrasound image of the tumors from the skin surface to its deepest part (after tumor “debulking” and referred to as ‘tumor skin’), and we obtained the following results: micronodular tumors now measured 1585 µm (SD = 332.3), nodular tumors measured 1473.8 µm (SD = 450.3), infiltrative tumors measured 1352.1 µm (SD = 570), and superficial tumors measured 584 µm (SD = 267.9). (Table 3) After tumor debulk, there was still a significant difference in tumor depth between different BCC subtypes, F (4,94) = 20.24, *p* = 0.000 (Table 4B).

**Figure 3:**
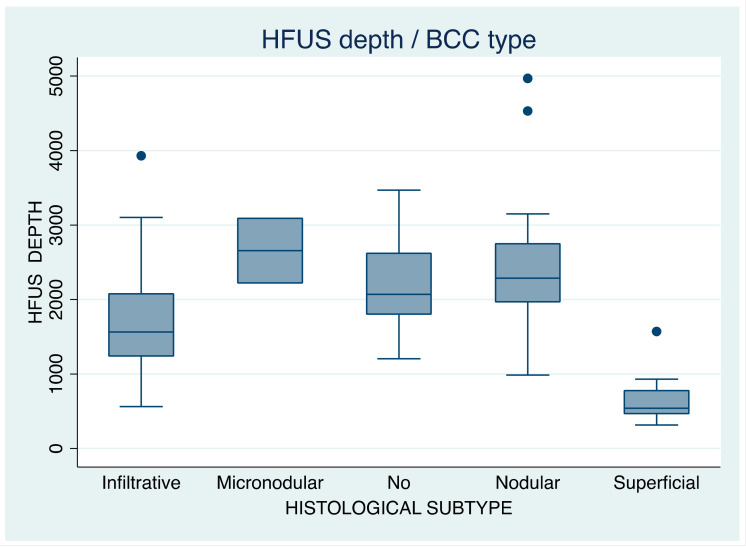
Box plot of total tumor depth (*Y* axis, before tumor “debulking”, HFUS depth), in µm, in relation to BCC histological subtype (*X* axis). ‘No’ in the *X* axis corresponds to ‘not available histology report’.

**Figure 4:**
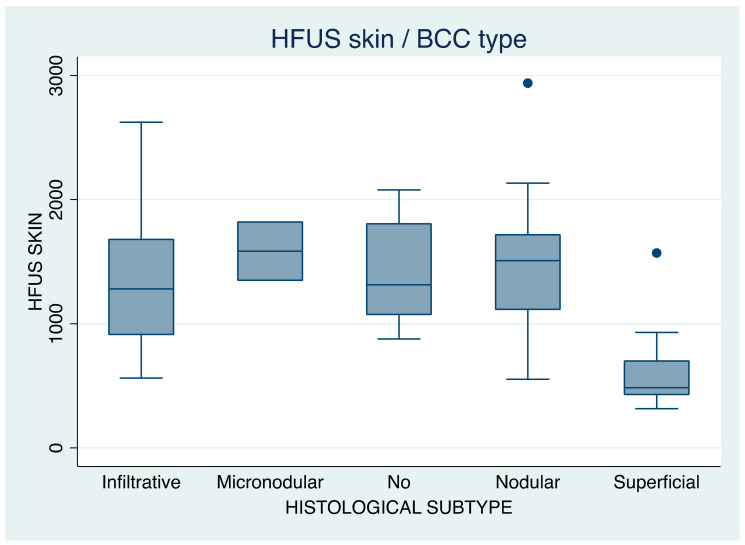
Box plot of tumor depth after tumor “debulking” (HFUS skin or tumor skin)*,* in µm, in relation to BCC histological subtype. ‘No’ in the *X* axis corresponds to ‘not available histology report’.

In both measurements (before and after tumor “debulking”), micronodular BCCs were found to be deeper than all other subtypes, followed by nodular, infiltrative, and, lastly, superficial ones. HFUS measurement results are presented in Table 3 and have also been graphically displayed in the two box plots (Figure 3 and Figure 4).

## 4. Discussion

Basal cell carcinomas are the most frequent skin malignancies. It is important to have non-invasive imaging tumor information that allows the therapeutic decision that will result in clinical cure with minimal treatment to be made. Ideally, we should be able to identify high-risk tumors that require surgical treatments [14], and treat low-risk tumors with minimally invasive techniques. Clinical information is not sufficient to take this decision. Dermoscopy is a non-invasive tool used in BCC diagnosis. However, HFUS provides additional important information: through ultrasound, we can visualize the shape, the length, and, most importantly, the depth of the tumors, as well as identify other tumor traits (i.e., hyperechoic granules) correlated with specific BCC subtypes.

BCC is generally confirmed by histopathological examination. However, skin biopsy provides information solely for the site where the biopsy has been taken. If a tumor is comprised of two different subtypes (nodular and superficial) and an incisional biopsy is performed in the area of the superficial BCC, the physician might incorrectly decide to use a non-invasive technique to treat the whole tumor, and the result of the treatment will probably be that the nodular part will only be partially treated. HFUS can be used as a quick, non-invasive technique to achieve three things. (1) To choose the biopsy site(s). Visualizing the whole tumor (especially important in large tumors) with the use of HFUS enabling us to detect deeper areas, and, as a result, to biopsy higher risk areas. (2) To choose the correct treatment modality. If the entire tumor is superficial, it can be effectively treated with PDT or imiquimod or cryosurgery. (3) To monitor the tumor area after treatment. In our study, high-frequency ultrasound imaging using a 22 MHz probe was performed on 100 BCC lesions in 54 patients, and the depth measured from the ultrasonographic image was analyzed and later related to the histological type.

The HFUS measurements correlated with the histology, and, indeed, micronodular tumors were found to be deeper than all other subtypes, followed by nodular, infiltrative, and, lastly, superficial ones. This correlation and order in the thickness of the tumor did not change, even when the protruding part of the tumor was removed from the measurement. Crisan et al. [6] have shown a significant correlation between U/S and histological findings regarding tumor thickness, pointing at the small differences caused by US overestimation, which may be explained by the presence of the perilesional tumoral infiltrate and/or the retraction of tissue induced during the excision of the tumor.

In our study, all 100 BCCs had shapes that could be easily described as irregular, ribbon, or round. The tumor shape, as visualized by the HFUS, seems to be predictive of the subtype: irregular-shaped tumors were more likely to be infiltrative BCCs, ribbon-shaped tumors were more likely to be superficial BCCs, and round-shaped tumors were likely to be nodular BCCs. When superficial BCCs were described as round shaped in the HFUS, they were thinner (less than 400 microns). In all cases, we were able to visualize the morphology, exact localization, and thickness in a reliable way before surgery, and we could thus also perform a correlation with the histological type described after excision. The sonographic appearance of the tumors studied (hypoechoic and oval shaped) was similar to previous reports in the literature [15].

Furthermore, the depth of the lesions displayed by ultrasound has been helpful for the differential diagnosis of lesions at different risk levels. Wand et al. [13] showed that all high-risk BCC lesions examined (defined as micronodular, infiltrative, basosquamous, and mixed) involved the sub-cutaneous tissue, while 78% of low-risk lesions (defined as superficial and nodular) were located in the dermis, resulting in a significant difference between the two groups. The authors concluded that pre-operative ultrasound can be employed to reveal subclinical characteristics of the tumor, which can be crucial to providing important information for therapeutic decision making, and which can also predict the risk of recurrence.

As high-frequency ultrasound has been reported to explicitly present the deep structure of lesions and, in our experience, also measure the tumor depth that correlates with the histological type, important information can be obtained from this method for therapeutic decision making [4,5,6,15]. Similarly OCT will also provide information on both the morphology and the depth of a tumor. Although its resolution is more precise than that of the ultrasound, the cost of the machine is much higher. On the other hand, RCM has a high resolution, allowing for the examination of lesions at a cellular level, but it cannot penetrate deeply into the skin and, thus, it cannot provide information on the depth of a tumor whilst having, and, at the same time, it is also very expensive to purchase one. All of these diagnostic techniques may play an important role in basal cell carcinoma management, but they all require training to use them, and, moreover, the high cost of both OCT and RCM makes them unrealistic for daily office practice. These two techniques may thus be better reserved for use in academic centers and hospitals [16,17].

**Figure 5:**
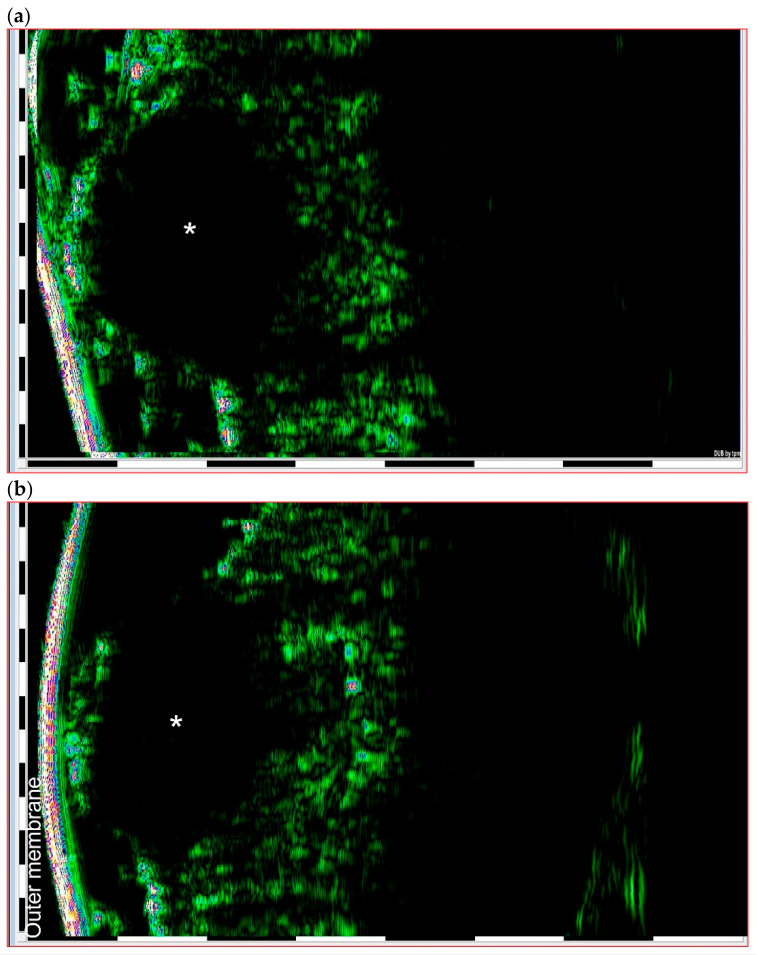
HFUS BCC images of a nodular BCC (round shape with well-defined margins) (**a**) before surgical excision (in vivo HFUS), and (**b**) the same tumor after excision (ex vivo HFUS). The star corresponds to the center of the tumor; the outer membrane is the film placed between the ultrasound head and the skin for protection.

Knowing the shape and depth of the tumor allows the surgeon to decide on whether to treat the lesion with minimally invasive techniques, such as shaving, curettage and electrocoagulation, photodynamic therapy, or cryosurgery. Once a tumor has been identified as small, it is possible to just shave it and visualize it ex vivo in order to guarantee that it has been all removed (Figure 5) [18,19].

Furthermore, correct evaluation of surgical margins is of paramount importance, especially in regards to infiltrative BCCs, which pose a challenge when assessing their margins pre-operatively, resulting in higher rates of incomplete surgical excision [20,21]. HFUS can improve the assessment of basal cell carcinoma margins preoperatively [18]. In our study, all infiltrative BCCs showed clear margins in the pathology report after excision.

In addition, we found numerous intra-tumoral hyperechoic granules in two tumors that were histologically described as micronodular. This finding corresponds with previous descriptions found in the literature by Worstman et al., which stated that hyperechoic spots may predict the high-risk BCC subtypes [22].

Bobadilla et al. [15] studied the capacity of ultrasound in defining a sonographic morphology for BCC, and they tried to ascertain the accuracy level of the measurement of the unknown axis (depth) of the lesion. They argued that ultrasound is indeed helpful when setting up BCC surgery because it can discern, in a non-invasive way, lesions and their depth as well as the patterns of the vessels. Additionally, ultrasound has a fine thickness correlation with histology, and it can also uncover subclinical satellite lesions. Thus, it appears that ultrasound is an interesting approach not only for pre-surgical evaluation but even possibly for follow-up of lesions that are treated with non-invasive treatments.

In conclusion, high-frequency ultrasound appears as a reliable technique to provide important information for pre-operative evaluation of BCC, helping physicians to decide on the optimal therapeutic approach and tailor the treatment to the characteristics of the lesion.

## 5. Conclusions

The 22 MHz high frequency ultrasound (HFUS) is a non-invasive imaging technique that gives valuable subclinical tumor information, such as depth, length, volume, and shape. It is a relatively simple procedure that gives valuable preoperative information, which helps personalize the treatment and reduce over- or under-treatment. The level of invasion of a NMSC is usually known only after the tumor has been removed and after the histopathological report has been received. HFUS provides this information pre-operatively and can help to guide therapeutic decisions. For instance, the physician may decide to treat thin tumors with minimally invasive techniques and reserve more aggressive treatments for deeper tumors. We have been able to show an association between BCC histological subtype and tumor depth and shape. We calculated an unweighted Cohen’s Kappa statistic coefficient equal to 0.8251 (se 0.0735, z [for k = 0] 11.23, *p* < 0.0001), which indicates an almost perfect agreement beyond chance between BCC subtypes evaluated by histological examination and U/S appearance [23]. The latter highlights the significance and diagnostic accuracy of HFUS as a non-invasive pre-operative diagnostic tool. Our study’s limitations derive mainly from its retrospective, non-controlled, and non-randomized design. Larger well-designed series are still needed to explore the role of HFUS in shaping BCC management strategies.

## Figures and Tables

**Table 1:** Population statistics. (*n* = 100). The table shows the distribution of various characteristics (gender, age, tumor location, histological subtype, tumor shape, and margins as obtained by the HFUS) within the dataset.

Variable	Value	Frequency (%)	Mean (SD)	Median (IQR)
Gender	Male	66 (66.0)		
	Female	34 (34.0)		
Age			73.1 (11.6)	71.0 (62–84)
Localization	Head	64 (64.0)		
	Neck	2 (2.0)		
	Upper limb	5 (5.0)		
	Lower limb	1 (1.0)		
	Torso	28 (28.0)		
Type	Superficial	29 (29.0)		
	Nodular	33 (33.0)		
	Micronodular	2 (2.0)		
	Infiltrative	21 (21.0)		
	Superficial + Infiltrative	1 (1.0)		
	Missing	14 (14.0)		
Shape	Ribbon	26 (26.0)		
	Round	46 (46.0)		
	Irregular	28 (28.0)		
Margins	Well-defined	74 (74.0)		
	Ill-defined	26 (26.0)		

**Table 2:** Cross-tabulations of 85 cases after excluding those cases where the histological subtype was not available (*n* = 14) and the one case of superficial plus infiltrative histological subtype (*n* = 1).

A. Cross-Tabulation between the Histological Subtype and the Tumor Shape (*n* = 85).					
Histological Subtype		Shape			Total
		Irregular	Ribbon	Round	
Infiltrative	Count	16	0	5	21
	% within subtype	76.2	0.0	23.8	100.0
	% within shape	69.6	0.0	13.5	24.7
Micronodular	Count	0	0	2	2
	% within subtype	0.0	0.0	100.0	100.0
	% within shape	0.0	0.0	5.4	2.4
Superficial	Count	0	25	4	29
	% within subtype	0.0	86.2	13.8	100.0
	% within shape	0.0	100.0	10.8	34.1
Nodular	Count	7	0	26	33
	% within subtype	21.2	0.0	78.8	100.0
	% within shape	30.4	0.0	70.3	38.8
Total	Count	23	25	37	85
	% within subtype	27.1	29.4	43.5	100.0
	% within shape	100.0	100.0	100.0	100.0
**B. Cross-Tabulation between the Histological Subtype and the Tumor Margins (*n* = 85).**					
**Histological Subtype**			**Margins**		**Total**
			Well Defined	Ill Defined	
Infiltrative		Count	11	10	21
		% within subtype	52.4	47.6	100.0
		% within margins	18.0	41.7	24.7
Micronodular		Count	1	1	2
		% within subtype	50.0	50.0	100.0
		% within margins	1.6	4.2	2.4
Superficial		Count	24	5	29
		% within subtype	82.8	17.2	100.0
		% within margins	39.3	20.8	34.1
Nodular		Count	25	8	33
		% within subtype	75.8	24.2	100.0
		% within margins	41.0	33.3	38.8
Total		Count	61	24	85
		% within subtype	71.8	28.2	100.0
		% within margins	100.0	100.0	100.0

A. Pearson chi^2^(6) = 94.4718 *p* = 0.000. B. Pearson chi^2^(3) = 6.351 *p* = 0.096.

**Table 3:** HSUF measurements, in μm.

*HFUS Measurements* (*n* = 100)	Category	Count (%)	Mean Value (SD)	Median (IQR)
**HFUS depth**			1702.6 (992.6)	1742 (801–2266)
**HFUS skin**			1180.8 (572.3)	1156 (656–1596)
**HFUS length**	<2500	0 (0.0)		
	2500–5000	8 (8.0)		
	5001–7500	39 (39.0)		
	7501–10,000	29 (29.0)		
	10,001–13,000	22 (22.0)		
	>13,000	2 (2.0)		
***Measurements by BCC subtype (n = 85, excl missing and mixed type histology)***				
**HFUS depth**	Superficial		620.6 (255.5)	539 (469–776)
	Nodular		2413.4 (798.2)	2286.5 (1969–2749)
	Micronodular		2656.5 (613.1)	2656.5 (2223–3090)
	Infiltrative		1692 (791.2)	1563 (1242–2076)
**HFUS skin**	Superficial		584 (267.9)	485 (430–700)
	Nodular		1473.8 (450.3)	1508 (1116–1717)
	Micronodular		1585 (332.3)	1585 (1350–1820)

**Table 4:** One-way ANOVA table. The table assesses whether there are any statistically significant differences between the means of tumor depth for the various BCC subtypes.

A. Before Tumor ‘Debulking’.					
Source	SS	df	MS	F	Prob > F
Between groups	55,382,578.5	4	13,845,644.6	32.05	0.0000
Within groups	40,179,899.5	93	432,041.93		
Total	95,562,478	97	985,180.186		
**B. After Tumor ‘Debulking’.**					
**Source**	**SS**	**df**	**MS**	**F**	**Prob > F**
Between groups	14,854,538.9	4	3,713,634.72	20.24	0.0000
Within groups	17,247,804.5	94	183,487.282		
Total	32,102,343.4	98	327,574.933		

A. Bartlett’s test for equal variances: chi^2^(4) = 30.3928 Prob > chi^2^ = 0.000. B. Bartlett’s test for equal variances: chi^2^(4) = 12.3506 Prob > chi^2^ = 0.015.

## Data Availability

Data are available upon reasonable request from the corresponding author. All data relevant to this study are included in the article.
